# Effect of Local Administration of Vancomycin to the Wound on Renal and Hepatic Function After Cardiac Surgery in Neonates

**DOI:** 10.3390/diseases14030093

**Published:** 2026-03-04

**Authors:** Vitaliy V. Suvorov, Davlet B. Sayitkuliev

**Affiliations:** Department of Surgical Diseases of Children, Saint-Petersburg State Pediatric Medical University, 194100 Saint-Petersburg, Russia

**Keywords:** local antibiotics, vancomycin, topical administration of vancomycin, sternal infection, mediastinitis prophylaxis, prevention of sternal infection

## Abstract

The development of sternal infection in neonates after cardiac defect correction using median sternotomy is a serious complication, increasing the length of hospital stay, mortality, and treatment costs. One effective method for preventing this complication is the local administration of antibiotics to the wound. The objective of this study was to evaluate the effect of local antibiotic application on renal and hepatic function in the postoperative period. **Methods**: A retrospective analysis of the treatment of 130 newborns with congenital heart defects (CHDs) was conducted. A local antibiotic (vancomycin, 0.5–1 g) was administered to the wound during sternotomy closure to prevent sternal infection. Liver and kidney function were assessed based on changes in serum alanine aminotransferase (ALT), aspartate aminotransferase (AST), and creatinine levels preoperatively and at 1 and 3 days postoperatively. Data were analyzed using repeated-measures analysis of variance (ANOVA) and Friedman’s chi-square test. **Results**: In total, local vancomycin was administered to the wound during sternotomy closure in 130 newborns after the correction of CHDs. Thirty-three patients were excluded from the study because intraoperative signs of acute kidney injury were noted. Thus, 97 newborns were included in the study and there were no cases of sternal infection in this cohort of patients. According to the results from the statistical data analysis, the preoperative ALT level was lower (Md = 19.2) than the postoperative ALT level on the first day (Md = 23, *p* = 0.076). On the third day of postoperative observation, after the local application of vancomycin, the ALT level increased slightly, but remained within the normal range (Md = 26, *p* < 0.001). The AST level on the first day was higher (Md = 43.2) than the preoperative AST level (Md = 39, *p* = 0.002). However, on the third day after surgery, the AST level decreased (Md = 36.4, *p* = 0.059) and remained within the normal range. The differences in the dynamics of ALT levels on the third day and AST on the first day after surgery were statistically significant. These levels corresponded to normal levels, leading to the conclusion that the local application of vancomycin has no effect on the levels of AST and ALT. On the first day after surgery, creatinine values were lower (M = 58.3) than before (M = 62.3, *p* = 0.073). On the third day of postoperative observation, the creatinine values were lower than before surgery (M = 56.8, *p* = 0.009). Creatinine levels decreased after the local application of vancomycin. **Conclusions**: The use of vancomycin locally in the wound intraoperatively in newborns after CHD repair did not result in a clinically significant increase in ALT, AST, or creatinine in the blood plasma in the early postoperative period, proving that there were no negative effects on renal and hepatic function during three postoperative days.

## 1. Introduction

Infectious complications in newborns result in prolonged hospitalization, elevated risk of severe conditions and mortality rate, and a significant increase in patient treatment costs [1,2]. Sternal infection in cardiac surgery is one such complication. Sternal infection is often caused by Gram-positive flora: Staphylococcus aureus or Staphylococcus epidermis [3,4]. According to numerous studies, one variety of these bacteria is methicillin-resistant Staphylococcus aureus (MRSA), which occurs in one-third of cases [5,6]. Mortality rates are higher if the pathogen in a sternal infection is MRSA, so it is necessary to adhere to strategies for the prevention of sternal infection after cardiac surgery, especially in high-risk patients. To reduce the incidence of sternal infection, local antibiotics can be used intraoperatively immediately before wound closure [7,8]. For example, in 1989, Vander Salm T.J. et al. used local vancomycin to achieve a reduction in the incidence of sternal infection [9].

With parenteral antibiotic administration, the concentration of the substance in tissues often depends on the time interval between the administration of the substance and the start of surgery. Damage to the skin allows microorganisms to spread into the wound. Any tissue damage is followed by inflammation, one of the purposes of which is to limit the penetration of substances and microorganisms from the wound into the bloodstream and vice versa [5,10]. Therefore, with parenteral antibiotic administration, achieving an effective therapeutic dose in the wound is a difficult task, as it is often necessary to administer a high dose of the drug. The local application of antibiotics to the wound avoids the risk of toxic effects on organ function from high doses and also promotes more effective prevention of wound infection. However, when using high doses of antibiotics locally, it is necessary to consider their possible impact on the function of excretory organs: the kidneys and liver [11]. In this regard, the aim of this study was to evaluate the effect of local vancomycin on liver and kidney function in neonates after cardiac surgery using median sternotomy.

## 2. Materials and Methods

### 2.1. Study Cohort and Study Design

The study was based on the results of the treatment of 130 newborns (age < 28 days) with congenital heart defects (CHDs) who underwent cardiac surgery via a longitudinal median sternotomy. A single-center, single-arm, retrospective cohort study was conducted. The local ethics committee of St. Petersburg State Pediatric Medical University approved the protocol for this study (No. 29/05; 7 August 2023). Patients’ parents gave their informed consent for the use of examination and treatment data in the scientific research.

The criteria for inclusion in the study were children with CHDs who underwent surgery with complete median sternotomy, who were aged from 0 to 28 days, and who received locally administered antibiotics (vancomycin) in a surgical wound. The criteria for exclusion from the study were the application of extracorporeal membrane oxygenation; renal and hepatic failure before surgery; signs of renal and hepatic failure intraoperatively; and concomitant pathology of the kidneys and liver.

A standardized protocol for the prevention of infection was used for all patients: second-generation cephalosporins (cefuroxime) were administered parenterally. The total antibacterial load dose was 150 mg/kg/day (in three doses). The first was administered intravenously at a rate of 50 mg/kg 2 h before surgery. The second and third doses of 50 mg/kg were administered intravenously at intervals of 6–8 h after surgery. The course was 3 perioperative days (150 mg/kg/day). After using cefuroxime, transient elevations in liver enzymes, including aspartate transaminase, alanine transaminase, is rarely observed [12,13,14].

**Figure 1:**
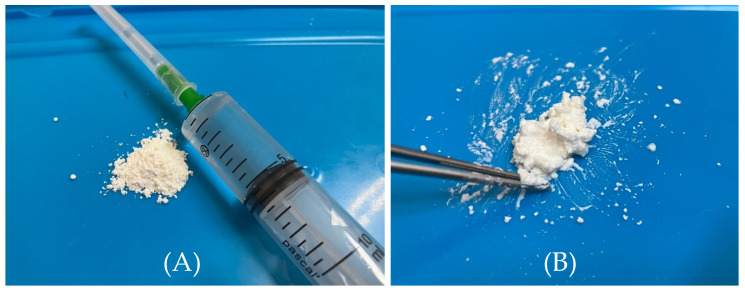
Making a paste from an antibiotic (vancomycin) for the surgical wound. (**A**) 0.5–1 g of vancomycin mixed with 2–4 mL of 0.9% NaCl solution. (**B**) The antibiotic (vancomycin) in the form of a paste.

All patients received a glycopeptide antibiotic—vancomycin—which was locally applied to the wound during sternotomy wound suturing. Vancomycin was applied topically as a “paste”: 0.5–1 g of vancomycin was mixed with 2–4 mL of 0.9% NaCl solution and applied to the sternal incision edges [10,15]. It was freshly prepared before being applied to the wound. Until a wound is completely sutured, it should be irrigated with antibiotic solution during soft tissue closure: 10 mL of 0.9% NaCl mixed with 0.5–1 g vancomycin [16] (Figure 1).

To assess potential liver and kidney damage, alanine aminotransferase (ALT), aspartate aminotransferase (AST), and creatinine levels in venous plasma were measured. Monitoring was performed preoperatively and postoperatively on days 1 and 3. Liver dysfunction was assessed by changes in ALT and AST levels. Normally, AST levels are no greater than 58 Units/L, and ALT no greater than 56 Units/L. Considering that the level of these enzymes may increase due to surgical intervention (damage to tissue, muscles, and heart), a significant increase in these values (more than twice the normal amount) may also indicate liver damage. ALT and AST levels were determined using the ultraviolet kinetic method, which measures enzyme activity. Samples were collected at the same time intervals after antibiotic administration.

Renal impairment was assessed based on changes in plasma creatinine levels before and after surgery. A 1.5–2-fold increase in creatinine levels in the postoperative period compared to the preoperative (baseline) level within 3 days was considered a criterion for the presence of acute kidney injury [17,18]. Creatinine levels were measured using an enzymatic method.

### 2.2. Statistical Analysis

To determine the degree of possible toxic effects of the antibiotic, a comparative analysis of occurrence of renal and/or liver dysfunction in the postoperative period was performed using the repeated-measures analysis of variance (ANOVA) for continuous variables with normal distributions [19]. The level of statistical significance was accepted as *p* < 0.05. For abnormal distributions, Friedman’s chi-square test was used. When statistically significant differences were identified, the groups were compared pairwise using the Wilcoxon signed-rank test with the Bonferroni correction (*p* < 0.025).

To evaluate the impact of risk factors on the development of acute renal failure after surgery, we performed multivariate logistic regression analysis. The level of statistical significance was accepted as *p* < 0.05. To perform the analysis, the influence of the following risk factors was assessed: height, weight, sex, age at the time of surgery, genetic abnormalities, systemic hypoxemia before surgery, level of oxygen delivery and hematocrit before surgery, poor systolic function of the left ventricle (or systemic) before surgery, preoperative mechanical ventilation, type of CHD, risk according to the RACHS (risk adjustment for congenital heart surgery) scale, type of operation, use of cardiopulmonary bypass and duration, level of intraoperative hypothermia, and duration of surgery. Statistical processing was carried out using SPSS for Windows (Version 25.0, IBM Corp., Armonk, NY, USA).

## 3. Results

A local antibiotic (vancomycin) was administered to wounds during sternotomy closure to prevent sternal infection in 130 newborns after the correction of CHDs. Intraoperative signs of acute kidney injury were noted in 33 patients, and peritoneal drainage was performed for dialysis. Those patients were subsequently excluded; thus, 97 newborns were included in the study.

The mean age of the 97 operated newborns at the time of surgery was 11.4 days (minimum—0 days, maximum—28 days). The mean weight was 3.1 kg (minimum—1.2 kg, maximum—4.8 kg). Overall, there were no cases of sternal infection. Sternal closure was delayed in 25 (25.8%) patients who were clinically unstable at the end of the surgery. These patients had D-TGA (*n* = 12), TAPVR (*n* = 3), single-ventricle physiology (*n* = 7), and status post arch repair (*n* = 3) because of severe coarctation with hypoplastic aortic arch. A more detailed description of the patients is presented in Table 1 and Table 2.

Friedman’s chi-square test was used for ALT and AST. Statistically significant differences were found in both groups. The levels of ALT and AST in blood plasma on the first and third control days after the local application of vancomycin changed statistically significantly: χ^2^_F(2)_ = 13.865 (*p* = 0.001) and χ^2^ _F(2)_ = 8.333 (*p* = 0.016). To identify these differences, pairwise comparisons were made between groups using the Wilcoxon signed-rank test with a Bonferroni correction. According to the results of the statistical data analysis, the preoperative ALT level was lower (Md = 19.2) than the postoperative ALT level on the first day (Md = 23) by an average of 3.8 Units/L (*p* = 0.076). On the third day of postoperative observation after the local application of vancomycin, the ALT level increased slightly, but remained within the normal range (Md = 26, *p* < 0.001). The AST level on the first day after the local application of vancomycin was higher (Md = 43.2) than the preoperative AST level (Md = 39, *p* = 0.002). However, on the third day after surgery, the AST level decreased (Md = 36.4, *p* = 0.059) and remained within the normal range, and there was no clinically significant effect of local application of vancomycin on the levels of AST and ALT (Table 3).

**Figure 2:**
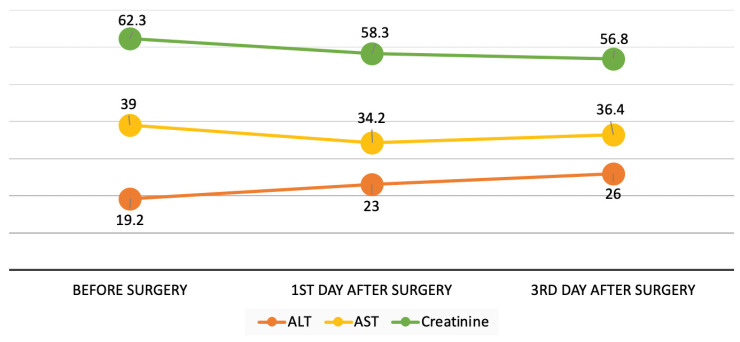
Schematic representation of the dynamics of changes in levels of ALT, AST, and creatinine.

The required condition of sphericity was met (Mauchly’s χ^2^_(2)_ = 1.359, *p* = 0.507). Statistically significant differences were found between the mean values of plasma creatinine levels on the first and third days after the topical application of vancomycin: F(1.97, 189.3) = 3.63, *p* = 0.029. On the first day after surgery, the creatinine values were lower (M = 58.3) than before surgery (M = 62.3) by an average of 4.1 μmol/L (*p* = 0.073). On the third day of postoperative observation, the creatinine values were lower (M = 56.8) than before surgery by an average of 5.5 μmol/L (*p* = 0.009) (Table 4). The fact that creatinine levels decreased after the local application of vancomycin indicates that there are no adverse systemic effects on renal function (Figure 2).

The serum creatinine levels were elevated in most patients before surgery, but there were no clinical signs of impaired renal function. Taking into account the high proportion of intraoperative signs of acute renal failure, 33 patients were excluded from the study. The influence of pre- and intraoperative risk factors on the development of acute renal failure was analyzed. As a result, multivariable regression analysis identified only one a statistically significant risk factor associated with the development of renal failure in neonates after cardiac surgery—the level of oxygen delivery in the preoperative period (OR = 123.75, CI: 11.588–1321.574, *p* < 0.001), which increased this risk by  123 times.

## 4. Discussion

One effective method for preventing wound infection is the local application of antibiotics to the wound [20,21,22,23,24,25]. Currently, there is a limited number of scientific publications devoted to this topic in pediatric practice [26].

Vancomycin is a nephrotoxic drug, with clinical manifestations of acute kidney injury developing dose-dependently, especially in patients with risk factors for kidney damage. In this situation, it is difficult to determine the direct role of vancomycin in the development of this complication. In this study, we analyzed the development of signs of kidney and liver injury in neonates in the early postoperative period who received local application of vancomycin in the sternotomy wound to prevent sternal infection.

In the present study, we analyzed the dynamic change in the concentrations of creatinine, ALT, and AST in venous blood plasma for three days after surgery. The creatinine values on the first day after surgery were lower than before, but without statistical significance (*p* = 0.073). However, on the third day, the creatinine level changed statistically significantly, but the clinical absolute values remained within the neonatal reference range and were low (M = 56.8, SD = 19.3, *p* = 0.009). The ALT levels were lower before surgery than on the first day after surgery (*p* = 0.076). However, on the third day of postoperative observation, the ALT levels increased slightly but remained within the normal range (*p* < 0.001). The AST level on the first day was higher than the preoperative level (*p* = 0.002), but on the third day after surgery it decreased (*p* = 0.059) and remained within the normal range. Despite the fact that the differences in the dynamics of ALT levels on the third day and AST on the first day after surgery were statistically significant, these levels corresponded to normal values. Therefore, no signs of a clinically significant increase in creatinine levels or impaired renal function were detected in the study group of newborns during the three postoperative days. There was also no clinically significant effect on changes in the levels of these liver enzymes during this period.

This antibiotic does not penetrate tissue well, so higher doses are often required when administered systemically, which increases the risk of organ toxicity, primarily affecting the kidneys. With parenteral administration, higher concentrations of the drug accumulate in the kidneys compared to other organs [27,28]. Approximately 80–90% of vancomycin is excreted primarily by the kidneys in unchanged form. As a result of the intracellular accumulation of the drug during its endocytosis in the tubular cells of the kidneys, damage to the epithelium of the proximal tubules occurs. This pathogenetic mechanism of kidney injury subsequently leads to oxidative stress, accompanied with the development of inflammation and cellular damage, mitochondrial dysfunction, and, ultimately, cellular apoptosis in the proximal renal tubules [28,29,30].

When vancomycin is applied locally, the absorption of the drug into the bloodstream depends not only on the local concentration of the drug, but also on local conditions in the wound: the level of contamination, the presence of wound secretions, the severity of tissue edema, and vascularization. All of these conditions primarily provide a barrier that helps reduce the degree of penetration of the antibiotic from the bloodstream into the wound and from the wound into the systemic bloodstream. Therefore, to ensure an effective level of vancomycin in the tissues of the postoperative wound, local application is preferable.

The results of several recent systematic reviews with meta-analyses in various fields of surgery show that the local application of vancomycin and other antibiotics to the wound at the suturing stage effectively reduces the risk of infection in clean surgical wounds [20,21,23]. Furthermore, the local application of antibiotics allows for high concentrations of the substance to be achieved in the wound [31]. At the same time, the systemic concentration of the antibiotic used remains low, and the systemic toxic effect on organs is reduced [32,33]. For example, in traumatology and orthopedics, this method of wound infection prevention has demonstrated high efficacy [22,34,35]. In a study by S.W. Young et al., a comparative analysis of the effectiveness of achieving vancomycin concentrations with intraosseous administration for the prevention of surgical infection during knee arthroplasty was conducted. Based on the result of the study, the authors concluded that during primary knee arthroplasty, the prophylactic use of low doses of vancomycin via intraosseous regional administration allowed for local tissue concentrations of the drug 5–20 times higher than with systemic intravenous administration to be achieved, despite the lower dose [35]. In 2025, R. Stauss et al. confirmed the high efficiency of the local application of vancomycin in knee arthroplasty and the absence of adverse systemic side effects, because the concentration of the antibiotic did not reach therapeutic values in the blood plasma in any patient [36].

Similar findings were obtained in a study of an adult cohort of cardiac surgery patients undergoing coronary artery bypass grafting. Fourteen patients undergoing elective coronary artery bypass grafting were given topical vancomycin (500 mg, prior to sternal suturing) to prevent sternal infection. Subsequently, serum vancomycin concentrations were monitored for several hours. Peak concentrations were observed at 30 min with a mean value of 2.96 mg/L (range, 0.99 to 5.00 mg/L). This blood level remained elevated for 6 h after surgery and was detectable in urine for 5 days after surgery [37].

In their review of the scientific literature, P.L. Magro et al. assessed the effectiveness of topical antibiotics for the prevention of sternal infection in adult patients. They concluded that topical antibiotics prevent the development of sternal infection. The most effective results were observed with gentamicin–collagen sponges and topical vancomycin [38].

In 2025, M.T. Tsai et al. analyzed the dynamics of achieving a therapeutic dose of vancomycin in cardiac surgery patients with renal failure [39]. The study included 129 adult patients. Before suturing the sternotomy wound, vancomycin (2.5 g) was applied topically to the sternal edge. Plasma drug concentrations were measured from day 0 to day 7 after surgery. The authors noted that plasma exposure of vancomycin after topical application to a wound depends primarily on renal function and body surface area. According to their data, in patients with end-stage renal failure who were on hemodialysis with BSA < 2 m^2^, a more frequent (20–30%) increase in vancomycin concentration above 10 mg/L was observed. Among patients without renal impairment, this probability was low [39].

Cardiac surgery patients are prescribed numerous other medications during treatment, in addition to vancomycin, that can negatively impact renal function. These include aminoglycoside antibiotics, antiviral drugs (such as acyclovir), inotropic agents (including vasopressors), and intravenous contrast agents used in X-ray diagnostics and treatment as well as during computed tomography and magnetic resonance imaging. Furthermore, loop diuretics and renin–angiotensin system blockers are required for heart failure therapy in almost all patients [40]. To determine the degree of effect of each drug in such a cohort of patients, a study with multivariate analysis is necessary.

In addition to nephrotoxicity, vancomycin has a low risk of hepatotoxicity. Similar to nephrotoxicity, the mechanism of hepatotoxicity is also associated with oxidative stress and mitochondrial dysfunction [33]. The reason for the significant difference between vancomycin nephrotoxicity and hepatotoxicity may be related to the different types of transporters in liver and renal tubular cells. Some studies have reported minor toxic effects after the systemic administration of vancomycin [41]. A low correlation between the development of a hepatotoxic effect and the use of vancomycin has been noted, which is confirmed by an increase in the levels of aspartate aminotransferase, alanine aminotransferase, and alpha-fetoprotein in only 0.37% of patients.

Thus, the local application of vancomycin is not only a highly effective way to reduce the risk of surgical infection, including mediastinitis, but is also the method associated with minimal systemic side effects.

### Limitations

When acute kidney injury occurs after cardiac surgery with cardiopulmonary bypass, it is difficult to determine the exact cause. In this case, it is also challenging to identify whether topical vancomycin is associated with the development of kidney injury. For example, decreased diuresis is possible due to a decrease in the volume of circulating blood, which is a transient disorder and is corrected by infusion therapy. Moreover, most nephrotoxic drugs are not oliguric.

It may also be necessary to study the delayed effects and include newborns with pre-existing kidney disease in the study. The specifics of the method used may also influence the occurrence of side effects associated with negative impacts on kidney and liver function. Consequently, further study of the pediatric cardiac surgery group with analysis of additional risk factors for the development of renal and hepatic dysfunction is required.

## 5. Conclusions

The topical intraoperative use of vancomycin in neonates after CHD correction did not result in a clinically significant increase in plasma ALT, AST, or creatinine levels in the early postoperative period. This finding suggests the absence of renal and hepatic dysfunction.

## Figures and Tables

**Table 1:** Demographic and clinical characteristics of the patients.

Characteristics	All Patients *	Study Group **	Excluded Group
Total number of patients, (n/%)	130/100	97/100	33/100
Height, cm (M)	50.6 [51; 62]	50.2 [42; 58]	51.2 [44; 58]
Weight, kg (M)	3.2 [1.2; 4.8]	3.1 [1.2; 4.8]	3.4 [1.5; 4.2]
Sex (male), (n/%)	81/62.3	59/60.8	22/66.7
Age at the time of surgery, days (M)	11 [0; 28]	11.4 [0; 28]	9.8 [0; 27]
Type of CHD, (n/%):			
- Coarctation of the aorta + arch hypoplasia/IAA	19/14.6	16/16.5	3/9.1
- D-TGA/D-TGA + VSD	16/12.3	12/12.4	4/12.1
- VSD/AVSD/TF/DORV	17/13.1	16/16.5	1/3
- Truncus arteriosus	9/6.9	6/6.2	3/9.1
- HLHS/Single ventricle	58/8.6	39/40.2	19/57.6
- TAPVR	4/3.1	3/3.1	1/3
- Other	7/5.4	5/5.2	2/6.1
Cyanotic CHD, overall	66/50.8	47/48.5	19/57.6
Genetic abnormalities, overall	17/13.1	11/11.3	6/18.2

* 130 in total: 97 included in the study and 33 excluded. ** Study group: the patients had no intraoperative signs of acute kidney injury. Notes: AVSD, atrioventricular septal defect; CHD, congenital heart defect; DORV, double-outlet right ventricle; D-TGA, D-transposition of the great arteries; HLHC, hypoplastic left heart complex; IAA, interrupted aortic arch; TF, tetralogy of Fallot; TAPVR, total anomalous pulmonary venous return; VSD, ventricular septal defect.

**Table 2:** Perioperative characteristics of the patients.

Characteristics	All Patients *	Study Group **	Excluded Group
Total number of patients, (n/%)	130/100	97/100	33/100
Type of operation, (n/%):			
- Aortic arch reconstruction (+other)	26/20	21/21.6	4/12.1
- Arterial switch (+other)	15/11.5	11/11.3	4/12.1
- Valvuloplasty (+other)	4/3.1	3/3.1	1/3
- VSD or AVCD repair	3/2.3	1/1	2/6.1
- Bilateral PA banding/ PA banding	52/40	36/37.1	16/48.5
- Norwood/DKS	10/7.7	7/7.2	3/9.1
- Other correction of a single ventricle	15/11.5	14/14.4	1/14.4
- Truncus arteriosus repair	1/0.8	1/1	0/0
- TAPVR repair	4/3.1	3/3.1	1/3
RACHS, (n/%):			
1	0/0	0/0	0/0
2	1/0.8	0/0	1/3
3	78/60	57/58.8	21/63.6
4	40/30.8	32/33	8/24.2
5	0/0	0/0	0/0
6	11/8.5	8/8.2	3/9.1
Preoperative:			
Repeat sternotomy, overall	4/3.1	1/1	3/9.1
Systemic hypoxemia, overall	56/43.1	30/30.9	26/78.8
Level of DO_2_ < 520 mL/min/m^2^, overall	18/13.8	3/3.1	15/45.5
Level of hematocrit < 40%, overall	27/20.8	16/16.5	11/16.5
Poor systolic function of LV (systemic), overall	10/7.7	0/0	10/30.3
Mechanical ventilation, overall	45/34.6	24/24.7	21/63.6
Intraoperative:			
CPB, overall	69/53.1	52/53.6	17/51.5
Duration of surgery, min (M)	249 [85; 720]	241.7 [85; 720]	270.5 [105; 690]
Duration of CPB, min (M)	67.5 [0; 310]	62.2 [0; 310]	83.2 [0; 310]
Hypothermia intraoperative:			
- 14–20 degrees C	2/1,5	0/0	2/6.1
- 20–28 degrees C	22/16.9	16/16.5	6/18.2
- 28–34 degrees C	14/10.8	10/10.3	4/12.1
- Normothermic C	92/70.8	71/73.2	21/63.6
Hypothermic circulatory arrest, overall	8/6.2	3/3.1	5/3.1
Postoperative:			
Peritoneal dialysis, overall	33/25.4	0/0	33/100
Delayed sternal closure, overall	41/31.5	25/25.8	16/48.5
Postoperative bleeding, overall	5/3.8	3/3.1	2/6.1
Postoperative acute renal failure, overall	14/10.8	0/0	14/42.4
Duration of mechanical ventilation, days (M)	11.5 [1; 180]	8.3 [1; 38]	21 [3; 180]
ICU length of stay, days (M)	16.4 [2; 186]	12.3 [2; 47]	28.5 [5; 186]
Sternal infection, overall	1/0.8	0/0	1/3

* 130 total: 97 included in the study and 33 excluded. ** Study group: the patients had no intraoperative signs of acute kidney injury. Notes: AVSD, atrioventricular septal defect; CPB, cardio-pulmonary bypass; DKS, Damus–Kaye–Stansel procedure; DO_2_, oxygen delivery; ICU, intensive care unit; LV, left ventricle; PA, pulmonary artery; RACHS, risk adjustment for congenital heart surgery; TAPVR, total anomalous pulmonary venous return; VSD, ventricular septal defect.

**Table 3:** Perioperative liver enzyme levels in patients of the study group. Results of comparative analyses between the preoperative enzyme levels and the level on the 1st or 3rd postoperative day (Friedman’s chi-square test and Wilcoxon signed-rank test).

Friedman’s chi-square test					
Compared groups	χ^2^			F	*p*-value
ALT, preoperatively-on day 1-on day 3	13.865			2	0.001
AST, preoperatively-on day 1-on day 3	8.333			2	0.016
Wilcoxon signed-rank test					
Compared groups	Md_1_	Md_2_	T	z	*p*-value
ALT, preoperatively on day 1	19.2 [13.3; 33.6]	23 [16.2; 36.4]	1883	−1.776	0.076
ALT, preoperatively on day 3	19.2 [13.3; 33.6]	26 [18.8; 45.4]	1364	−3.644	<0.001
AST, preoperatively on day 1	39 [31.2; 55.2]	43.2 [34.6; 60]	1509.5	−1.12	0.002
AST, preoperatively on day 3	39 [31.2; 55.2]	36.4 [27; 43]	2679	−1.891	0.059

Notes: ALT, alanine aminotransferase; AST, aspartate aminotransferase; Md_1_—median of preoperative level, Md_2_—median of levels on the 1st or 3rd postoperative day at 25th and 75th percentiles.

**Table 4:** Perioperative kidney enzyme levels (creatinine) in patients of the study group. Results of comparative analyses between the preoperative creatinine level and the levels on the 1st or 3rd postoperative day (repeated-measures ANOVA).

Compared Groups	M_1_ (Sd)	M_2_ (Sd)	F	η^2^	*p*-Value
Preoperatively—on day 1	62.3 (23.2)	58.3 (16)	3.292	0.033	0.073
Preoperatively—on day 3	62.3 (23.2)	56.8 (19.3)	7.102	0.069	0.009

Notes: M_1_—mean of preoperative level, M_2_—mean of levels on the 1st or 3rd postoperative day.

## Data Availability

The datasets generated and/or analyzed during the current study are available from the corresponding author upon reasonable request.
